# Patience in Sickness

**Published:** 1890-05-10

**Authors:** 


### Words of Consolation.

### PATIENCE IN SICKNESS.

A sick person has above all others need of patience, and that of various kinds. Patience to bear pain and the privations of sickness, with its many heaped up trials. Patience with those around him, with their neglect, their misunderstandings, their stupidity. Patience to bear his own particular lot and the circumstances of his life. Above all, patience to wait the appointed time until his change cometh.

Like Job, he "has need of patience," and the need seems to grow sorer and sorer as time goes on. One day, perhaps, the difficulty of patience may be greatly lessened, the next the causes of impatience gather strength, the nerves are unstrung, everything tells on the sufferer. He tries to possess his soul in patience, but suddenly a fierce temptation comes, and he breaks down utterly. The impulse is only for a moment, but his sorrow of heart at having been betrayed into sin will long remain with him and take from him his confidence. It seems to him a hopeless thing to expect ever to become *habitually* patient, to *rest* in patience.

We are apt to look on patience as a natural gift, a thing belonging to some people which others are wholly without, and though it cannot be denied that some persons are certainly born with more natural patience than others, it will be of little use to them in the time of need. In a short illness it may avail, as the calls for patience are comparatively few, but when the weary days pass on, each bringing its own trials unguessed even by those around, there is felt the full need of the grace of patience. The sick soul sees he is naked and miserable and poor and blind, he can now understand why the Church prays for all sufferers, that they may have "patience under their sufferings," and feels what wonderful knowledge of his need has been shown in that prayer for patience. He must have it, or he cannot go on his weary way for an hour. He feels smitten and ashamed when some friend speaks of the patience with which he bears his trials. It looks like mockery to one whose life is spent in conflicts with impatience, whose groans and prayers are constantly going up to the God of patience, that He will make him like minded with Himself. If the friend is sincere, doubtless by the grace of God the struggles have been hidden from all human eyes, strength has been given to prevent the thoughts breaking forth into hasty words or deeds, and he may thank God and take courage.

